# Sample delivery for serial crystallography at free-electron lasers and synchrotrons

**DOI:** 10.1107/S205979831801567X

**Published:** 2019-01-09

**Authors:** Marie Luise Grünbein, Gabriela Nass Kovacs

**Affiliations:** a Department of Biomolecular Mechanisms, Max Planck Institute for Medical Research, Jahnstrasse 29, 69120 Heidelberg, Germany

**Keywords:** serial sample delivery, high-speed liquid jets, liquid injection, viscous matrices, high-viscosity extrusion, X-ray free-electron lasers, serial crystallography

## Abstract

The high peak brilliance and femtosecond pulse duration of X-ray free-electron lasers (XFELs) provide new scientific opportunities for experiments in physics, chemistry and biology. In structural biology, one of the major applications is serial femtosecond crystallography. The intense XFEL pulse results in the destruction of any exposed microcrystal, making serial data collection mandatory. This requires a high-throughput serial approach to sample delivery. To this end, a number of such sample-delivery techniques have been developed, some of which have been ported to synchrotron sources, where they allow convenient low-dose data collection at room temperature. Here, the current sample-delivery techniques used at XFEL and synchrotron sources are reviewed, with an emphasis on liquid injection and high-viscosity extrusion, including their application for time-resolved experiments. The challenges associated with sample delivery at megahertz repetition-rate XFELs are also outlined.

## Abstract 2

Current developments and challenges for serial sample delivery at synchrotrons and X-ray free-electron lasers are reviewed, including the new megahertz repetition-rate machines, with an emphasis on liquid injection and high-viscosity extrusion.

## 1. Introduction   

The unprecedented properties of X-ray free-electron lasers (XFELs) enable novel research in a variety of scientific fields (Pellegrini, 2016 ▸). An XFEL delivers as many photons in a single pulse (of femtosecond duration) as a third-generation synchrotron source does in an entire second. Moreover, the radiation is highly coherent (Pellegrini *et al.*, 2016 ▸). For macromolecular crystallography, this 10^9^-fold increase in peak brilliance means that high-resolution diffraction patterns can be collected from weakly diffracting objects such as tiny protein crystals (Colletier, Sawaya *et al.*, 2016 ▸; Gati *et al.*, 2017 ▸), including membrane proteins (Liu *et al.*, 2013 ▸). Importantly, the ultrashort pulse duration allows a diffraction pattern to be collected before the onset of radiation damage, even at room temperature (Neutze *et al.*, 2000 ▸; Chapman *et al.*, 2014 ▸). Thus, essentially damage-free data can be obtained even for radiation-sensitive samples (Young *et al.*, 2016 ▸; Suga *et al.*, 2017 ▸). XFELs also facilitate time-resolved (TR) measurements, including studies of irreversible reactions (Aquila *et al.*, 2012 ▸; Tenboer *et al.*, 2014 ▸; Nogly *et al.*, 2016 ▸; Nango *et al.*, 2016 ▸). The short pulse duration delivers temporal resolution down to the sub-picosecond range (Barends *et al.*, 2015 ▸; Pande *et al.*, 2016 ▸; Coquelle *et al.*, 2017 ▸). Also, the ability to use small crystals is crucial for both optically and chemically triggered reactions. Optical absorbance in a protein crystal is high, owing to the high protein concentration, and this limits optical penetration to at most a few micrometres (Barends *et al.*, 2015 ▸). In a similar fashion, the diffusion of chemical triggers into such crystals is often a rate-limiting step, with smaller crystals giving proportionally shorter diffusion times (Schmidt, 2013 ▸).

XFEL data collection, however, is complicated by the high intensity of the X-ray pulses, which ultimately destroy the sample (the formation of a diffraction pattern prior to destruction has been dubbed ‘diffraction before destruction’; Chapman *et al.*, 2014 ▸). Consequently, a fresh crystal (or crystal section) must be supplied for each exposure. A serial approach to data collection, termed serial femtosecond crystallography (SFX; Schlichting, 2015 ▸), becomes mandatory. SFX imposes unique challenges on all aspects of the experiment from sample preparation to sample delivery to data acquisition and analysis.

Most XFEL experiments require several thousands to many millions of homogeneously sized protein crystals. These are often prepared by batch-crystallization approaches (Kupitz *et al.*, 2014 ▸), including seeding to achieve homogeneity and control of crystal size (Nass *et al.*, 2016 ▸; Coquelle *et al.*, 2017 ▸; Dods *et al.*, 2017 ▸). Given the small size of the crystals, light microscopy may not suffice to characterize the samples, and alternative/complementary characterization techniques may be required. Second-order nonlinear optical imaging of chiral crystals (SONICC; Wampler *et al.*, 2008 ▸) can verify whether protein crystals are present in the sample, nanoparticle tracking analysis and dynamic light scattering can be used for crystal size measurements (Abdallah *et al.*, 2015 ▸; Darmanin *et al.*, 2016 ▸), screening of microcrystals at a synchrotron (Darmanin *et al.*, 2016 ▸) can provide information on crystal quality, and electron microscopy may serve all three purposes (Stevenson *et al.*, 2014 ▸).

The requirements for serial sample delivery into the X-ray interaction region are imposed by multiple factors that are dictated both by the experimental setup and by the properties of the sample. The sample must be reliably delivered at a constant rate to an interaction region defined by the X-ray focus of typically only ∼1–10 µm^2^ in cross-section (Liang *et al.*, 2015 ▸; Boutet *et al.*, 2016 ▸; Yabashi *et al.*, 2015 ▸). Since SFX experiments may be conducted either at low (Liang *et al.*, 2015 ▸) or ambient (Yabashi *et al.*, 2015 ▸; Boutet *et al.*, 2016 ▸) pressure, sample delivery should be spatially and temporally stable under both conditions. Sample transport must also be sufficiently fast so that pristine sample is present in the inter­action region for every X-ray pulse that can be recorded by the detector; otherwise the X-ray pulse-repetition rate must be lowered to match the rate at which fresh sample can be replenished. If the X-ray repetition rate is the limiting factor, this then increases the total time that is needed to collect a data set. Furthermore, sample delivery should be maximally efficient in terms of both hit rate (maximizing the fraction of recorded images that contain useful crystal diffraction) and sample consumption (maximizing the fraction of the sample probed by an X-ray pulse). Along with these technical requirements, sample delivery should be as gentle as possible in order not to alter the state of the sample under investigation. Neither the chemical and physical environment nor any mechanical stress arising from the transport process should change the structure, the charge or the electronic state of the specimen. This poses particularly strong constraints on delivery methods for biological (crystalline) samples, which are typically very sensitive to alterations of their environment, such as changes in solution chemistry, hydration, pressure or temperature. Simultaneously, the method must be compatible with the X-ray interaction and not give rise to undue background in the diffraction patterns that would mask weak high-resolution signals. Needless to say, while satisfying all of the above constraints, sample delivery should be as technically undemanding as possible.

Several serial sample-delivery methods have been developed over the last few years to address these complex experimental requirements. The challenge is lifted to a new level with the advent of XFEL sources operating at megahertz X-ray repetition rates, requiring much faster sample delivery, such as LCLS-II (Schoenlein, 2015 ▸) or the EuXFEL (Altarelli & Mancuso, 2014 ▸; Tschentscher *et al.*, 2017 ▸), with the first results from the latter having recently been published (Grünbein, Bielecki *et al.*, 2018 ▸; Wiedorn, Oberthür *et al.*, 2018 ▸).

Additionally, some of these sample-delivery techniques have been ported to synchrotrons, since in specific cases they can offer advantages over traditional synchrotron techniques. Numerous upgrades of existing synchrotron sources to diffraction-limited storage rings [DLSRs; for example SLS-II (Streun *et al.*, 2018 ▸), ESRF-EBS (Chenevier & Joly, 2018 ▸), PETRA IV (Wanzenberg *et al.*, 2017 ▸) and SPring-8 (Tanaka *et al.*, 2016 ▸)] and new DLSRs (Eriksson *et al.*, 2014 ▸; Rodrigues *et al.*, 2016 ▸) offer radiation with higher brightness (and coherence). Here serial data-collection approaches are particularly useful, as they spread the radiation dose across numerous separate crystals and thereby minimize radiation damage. SFX-derived sample-delivery techniques can be employed as they enable the convenient high-throughput collection of diffraction patterns from hundreds or thousands of small crystals at room temperature (Botha *et al.*, 2015 ▸; Nogly *et al.*, 2015 ▸; Stellato *et al.*, 2014 ▸; Roedig *et al.*, 2016 ▸; Martin-Garcia *et al.*, 2017 ▸; Weinert *et al.*, 2017 ▸) or cryo-temperature (Roedig *et al.*, 2015 ▸). TR measurements, including those of irreversible reactions, similar to TR-SFX, can also be performed, albeit with lower time resolution. To further increase the photon flux (∼100 times) and commensurately the time resolution, a pink synchrotron beam can be used instead of a monochromatic beam. This also works in the high-throughput serial data-collection regime, as successfully demonstrated by raster scanning a fixed target (Meents *et al.*, 2017 ▸).

In this review, we describe the sample-delivery techniques that have been developed over the last few years, with a particular focus on injection techniques. We discuss the underlying principles along with the specific advantages and challenges of each, especially for the technically more complex time-resolved experiments (for an overview, see Table 1 ▸). We also outline the challenges imposed on sample delivery by the novel megahertz X-ray repetition-rate XFELs and discuss how these can be addressed. The ultimate limits remain to be explored, however, as the initial experiments were performed at a relatively low irradiance (Grünbein, Bielecki *et al.*, 2018 ▸; Wiedorn, Oberthür *et al.*, 2018 ▸).

## 2. Injection   

Microcrystals suspended in their mother liquor or in a suitable carrier medium can be injected into the X-ray interaction region as a continuous jet or as droplets synchronized with the XFEL pulse. Different techniques for jet and droplet injection have been developed over the years, enabling the injection of samples of various viscosity ranges. Since these techniques operate in different flow regimes, they also present different opportunities and challenges (Weierstall, 2014 ▸) regarding sample efficiency, use at high repetition-rate XFELs and TR experiments.

### 2.1. Liquid injection   

**Figure 1:**
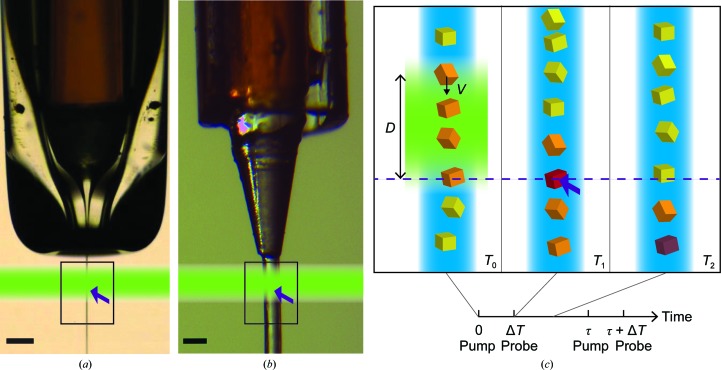
(*a*) GDVN injecting water. The sample is pumped through the inner capillary (brown), which is centred within the flame-polished larger capillary used to transport the sheath gas. The sample jet is focused by the sheath gas through the nozzle aperture, producing a micrometre-sized jet. (*b*) HVE injector injecting Vaseline. Again, the sample is transported through the inner capillary (brown) and the helium-gas stream flowing out of the outer capillary directs the extruded jet. In (*a*) and (*b*) the arrow indicates the X-ray interaction. For pump–probe experiments, a section of the sample jet is optically triggered (green shading) before X-ray probing. The black scale bar is 100 µm in length. (*c*) Constraints on time delays for time-resolved experiments valid for any injection system. The reaction is triggered in crystals (orange) within the segment hit by the pump pulse (optical axis indicated in green) at time *T*_0_. After a time delay Δ*T* the X-ray pulse (purple arrow) probes one of the excited crystals (red) at time *T*_1_. The jet speed *v* must be sufficiently low that crystals excited within the region *D* upstream of the X-ray optical axis have not yet all passed through the interaction region (dashed line), *i.e.* *v* < *D*/Δ*T*. All crystals triggered at *T*_0_ must clear the interaction region before the arrival of the subsequent probe pulse at *T* = Δ*T* + τ, where 1/τ is the X-­ray repetition rate, requiring the jet speed to be *v* > *D*/(τ + Δ*T*). This figure was adapted from Grünbein (2017 ▸).

The best-established XFEL injection method for protein crystals involves liquid (aqueous) jets formed via a gas-dynamic virtual nozzle (GDVN; Fig. 1 ▸ *a*; DePonte *et al.*, 2008 ▸; Weierstall *et al.*, 2012 ▸). GDVNs employ a sheath gas flow that focuses the liquid jet down to a diameter of typically 5 µm (Doak *et al.*, 2012 ▸). Jet diameters as small as a few hundred nanometres have been reported (DePonte *et al.*, 2009 ▸; Doak *et al.*, 2012 ▸). The principle of ‘gas focusing’ was originally demonstrated by Gañán-Calvo (1998 ▸) in a planar geometry, and was adapted by Doak and coworkers (DePonte *et al.*, 2008 ▸; Weierstall *et al.*, 2012 ▸) to a miniature tubular design suitable for XFEL operation. The latter was given the appellation GDVN to distinguish it from the very different Gañán–Calvo geometry. Gas focusing of the jet is critical for several reasons: (i) achieving comparable jet sizes with conventional Rayleigh jets without gas focusing would require physical nozzle apertures of approximately the jet diameter, which are highly prone to clogging (DePonte *et al.*, 2008 ▸); (ii) gas focusing allows stable jetting at relatively low flow rates of the order of 10 µl min^−1^ (Weierstall *et al.*, 2012 ▸); (iii) the small jet diameter reduces the X-ray scattering background from the carrier medium (Doak *et al.*, 2012 ▸), thus improving the signal-to-noise ratio as is crucial for weakly scattering samples; and (iv) for data collection in a vacuum the sheath gas prevents freezing of the jet (Gañán-Calvo *et al.*, 2010 ▸). Indeed, even when injecting into a vacuum the temperature of the GDVN microjet at the X-ray interaction region (typically ∼200 µm downstream of the nozzle tip) remains approximately that of the liquid solution within the injector (Gañán-Calvo *et al.*, 2010 ▸). Hence, measurements of biological samples near room temperature are possible even in a vacuum chamber. For most experiments, helium is used as a focusing gas owing to its inertness, high atomic speed and low X-ray scattering background.

A GDVN consists of a coned sample capillary of typically 50–100 µm inner diameter through which the sample suspension is pumped (Doak *et al.*, 2012 ▸). It is placed concentrically inside a larger capillary of converging shape that is used for transport of the sheath gas (Doak *et al.*, 2012 ▸). Typically, the converging shape of the sheath-gas capillary is obtained via manual flame-polishing (DePonte *et al.*, 2008 ▸). Given the inherent variability in GDVNs when fabricated in this manner, various other approaches have been developed to improve reproducibility: soft-layer lithography with polydimethyl­siloxane (PDMS) offers certain advantages here (Doak *et al.*, 2012 ▸; Trebbin *et al.*, 2014 ▸). Micro-injection moulding of ceramic nozzles can deliver satisfactory nozzles of high mechanical and chemical stability (Beyerlein *et al.*, 2015 ▸). 3D printing based on two-photon polymerization yields very high spatial resolution and an increased spectrum of accessible geometries in a limited total size of the printed object (Nelson *et al.*, 2016 ▸). Each of these techniques has its own challenges, but the automated fabrication, in principle, improves the reproducibility of fabrication. The known geometry of the fabricated GDVN can be used as a basis for fluid-dynamics simulations to assess the flow characteristics (Trebbin *et al.*, 2014 ▸) or, vice versa, the GDVN geometry can be optimized by means of such simulations. While nozzle-to-nozzle reproducibility is good, the inherent variability of manual fabrication also has advantages, specifically in that it can fortuitously lead to desired attributes that are not at all obvious and would not otherwise be explored.

GDVNs are typically run at a flow rate of 10–20 µl min^−1^ with pure water (Weierstall *et al.*, 2012 ▸) and up to a factor of two higher with actual sample suspensions. The speed and diameter of the emerging jet are determined (although not independently) by the nozzle geometry and by the pressure applied to the focusing sheath gas (Beyerlein *et al.*, 2015 ▸), which is controlled by a high-pressure gas regulator (Weierstall *et al.*, 2012 ▸). Depending on these parameters, in our experience and that of others the jet speed is typically in the range 10–50 m s^−1^ (Beyerlein *et al.*, 2015 ▸). To drive the sample solution through the injection system, a reservoir containing the microcrystal suspension is pressurized either with an inert gas or with a hydraulically driven piston. In the former case the sample flow rate is regulated by adjusting the pressure applied to the gas, while in the latter the hydraulic fluid (usually water) is supplied via a high-performance liquid chromatography (HPLC) pump (for example Shimadzu 20AD HPLC) and the HPLC flow rate is usually adjusted (Weierstall *et al.*, 2012 ▸). This has the advantage (insofar as elasticity in the sample and supply lines is negligible) that the flow remains constant even if the drive pressure must vary (for example owing to the possible accumulation of particulate material inside the lines). To counteract settling of the crystals within the sample reservoir during data collection it is mandatory that the reservoir is constantly rotated, which is best achieved using temperature-controlled antisettling devices (Lomb *et al.*, 2012 ▸). GDVNs generally require a minimum flow rate of ∼10 µl min^−1^ to generate a stable jet. At lower flow rates, the GDVN only ‘drips’ rather than ‘jets’ (DePonte *et al.*, 2008 ▸), which reduces the hit rate of the experiment dramatically. As a result of their moderate maximum repetition rates [120 Hz at LCLS (Emma *et al.*, 2010 ▸) and 60 Hz at SACLA (Ishikawa *et al.*, 2012 ▸)], the first-generation XFELs sampled only about one crystal per million in a GDVN jet (Beyerlein *et al.*, 2015 ▸). The jet flows continuously while the XFEL beam is pulsed, and the majority of the crystals pass through the interaction region unprobed in the intervals between XFEL pulses. Therefore, these experiments typically require several tens of milligrams of protein or more. To increase the efficiency of sample use and thereby reduce the sample consumption per data set, one must either decrease the flow rate of a continuous-flow jet (while nonetheless maintaining stable jetting conditions and the same hit rate), increase the data-collection rate of the XFEL or instead employ pulsed sample delivery that is synchronized with the X-ray pulses.

A reduction in flow rate with continuous jets can be achieved by employing a so-called double-flow focusing nozzle (DFFN), which is based on the same gas-focusing principle and geometry as a standard GDVN but employs an additional liquid-focusing stage prior to the final gas focusing (Oberthuer *et al.*, 2017 ▸). The sample jet is thus first focused by a sheath liquid, typically ethanol, and both liquids are then focused by the sheath gas. This allows the sample flow rate to be reduced to as low as 5 µl min^−1^ while still maintaining a stable jet (Oberthuer *et al.*, 2017 ▸). In addition to consuming less sample per data set, these double-flow focused nozzles exhibit a lower X-ray scattering background and seem to be less prone to accumulating salt and/or ice crystals at the nozzle tip, which improves their reliability during data collection (Oberthuer *et al.*, 2017 ▸). If longer jets are needed, vegetable oil (for example olive oil) can also be used as a sheath liquid, albeit at higher flow rates (Stricker *et al.*, in preparation).

Another approach to reducing the flow rate for stable continuous jetting is that of ‘electrospinning’. The microfluidic electrokinetic sample holder (MESH) uses an electric field of several kilovolts per centimetre to produce thin jets in this manner (Sierra *et al.*, 2012 ▸). MESH yields flow rates of only a few hundred nanolitres per minute, but requires glycerol or polyethylene glycol (PEG) to be present in (or added to) the sample suspension in order to extend the length of the jet before it breaks up into droplets (Sierra *et al.*, 2012 ▸). These additives also help to prevent freezing of the jet in a vacuum and to slow the settling of microcrystals in the sample-delivery lines, but limit the application of MESH to samples that are able to accommodate such carrier solutions (Sierra *et al.*, 2012 ▸). To avoid this complication, the concentric flow microfluidic electrokinetic sample holder (coMESH) was developed: similar to the double-flow focusing nozzle, the native sample suspension is injected concentrically into a sheath liquid [for example 2-methyl-2,4-pentanediol (MPD) or other cryoprotectants] used for electro-focusing, which again extends the length of the continuous jet and protects the sample against vacuum effects (Sierra *et al.*, 2016 ▸). The low sample flow rate of 1–3 µl min^−1^ allows the highly efficient collection of data from the injected sample (Sierra *et al.*, 2016 ▸).

#### 2.1.1. Intermittent sample delivery   

Sample consumption can also be reduced by delivering sample discontinuously into the X-ray interaction region, injecting a droplet or short jet segment only coincident with each XFEL pulse. Injection of droplets in the picolitre to nanolitre volume range (∼100 µm diameter) has been demonstrated by means of acoustic drivers (Ellson *et al.*, 2003 ▸; Roessler *et al.*, 2016 ▸) as well as piezoelectric drivers (Mafuné *et al.*, 2016 ▸). The timing between droplet ejection and XFEL pulse arrival needs to be controlled carefully, since the travel time of the droplet at a given speed from the nozzle tip to the interaction point must be taken into account (Roessler *et al.*, 2016 ▸). Given their intermittent mode of operation, the average flow rate of such droplet injectors can be quite low (∼0.5 µl min^−1^ for 30 Hz operation; Mafuné *et al.*, 2016 ▸). Although much less sample is consumed per data set relative to a continuously running GDVN, sample settling or buoyancy within the delivery system over time may lead to accumulation at preferred positions affecting the hit rate (Mafuné *et al.*, 2016 ▸) or droplet pinch-off (Roessler *et al.*, 2016 ▸). The generally large droplet diameter of pulsed injection results in a relatively high X-ray background, such that data are best collected from larger microcrystals that scatter more strongly (Roessler *et al.*, 2016 ▸). Acoustic droplet ejection has also been used to deliver small volumes of sample onto a fixed target (conveyor-belt drive) that is subsequently moved through the X-ray beam (Fuller *et al.*, 2017 ▸). This simplifies the task of placing a droplet into the interaction region at the very moment of the X-ray pulse, since timing is decoupled from the speed and the exact direction of droplet ejection. This approach is particularly interesting for TR experiments, especially with very long delays (approximately seconds), and facilitates the inclusion of complementary techniques such as X-ray emission spectroscopy (Fuller *et al.*, 2017 ▸).

#### 2.1.2. Mixing experiments with liquid jets   

The water-like viscosity of samples injected via a liquid jet allows the efficient chemical triggering of a reaction by turbulent or diffusive mixing with a small molecule prior to injection, a technique that is often referred to as ‘mix and inject’ (Schmidt, 2013 ▸). This is of particular interest since it allows intermediate structures of a reaction to be captured by probing the system with the X-ray pulse at a chosen time delay after initiation. The time resolution of such a measurement is determined by how fast the reaction can be initiated, *i.e.* by the time required for the substrate solution to intermix with the sample suspension and by substrate diffusion into the microcrystal (Schmidt, 2013 ▸), and by the transit time from the mixing zone to the X-ray interaction region. The latter depends on the fluid dynamics of the system (flow rate, flow geometry and viscosity; Calvey *et al.*, 2016 ▸). In any case, the reaction must be initiated very rapidly relative to the chosen time delay, otherwise a mixture of states will be observed (Schmidt, 2013 ▸).

To date, multiple types of mixing injectors have been developed, most of which include a GDVN-like gas-focusing stage to generate the micrometre-sized liquid jet that is ultimately probed by the XFEL pulse. For long time delays (of a few seconds or more), a simple mixing geometry suffices: using a T-junction far upstream (many centimetres to metres) of the injecting nozzle, sample and ligand are mixed at the T-junction and the reaction is allowed to proceed as the sample makes its transit into the X-ray interaction region. This type of mixing, combined with SFX data collection from a GDVN-generated liquid jet, has been used to capture structural intermediates of a riboswitch binding its ligand ∼10 s after reaction initiation (Stagno *et al.*, 2017 ▸) and of β-lactamase cleaving ceftriaxone at an ∼2 s time delay (Kupitz *et al.*, 2017 ▸). Similarly, using a different sample-delivery technique but the same mixing approach at a bright synchrotron source, the binding of lysozyme to its inhibitor chitotriose at time delays of 2 and 50 s was studied (Beyerlein *et al.*, 2017 ▸). While being a very simple and thus easy to set up mixing geometry, T-junction mixing is not suited for shorter time delays. Delay times based on transit through a capillary are intrinsically limited by the distribution of flow speeds across the capillary (for example Poiseuille flow, in which the flow speed is maximal along the capillary centreline and decreases to zero at the capillary wall; Batchelor, 1980 ▸). The transit time of a crystal travelling along the centreline of the capillary is therefore much less than that of a crystal travelling near to the wall, leading to an unavoidable spreading in delay times.

To probe short time delays (of a few milliseconds or less), the mixing process must take place close to the injector tip such that the travel time of the sample from the point of reaction initiation to the X-ray interaction region is short. Moreover, the effect of a non-uniform velocity profile on sample transport must be minimized downstream of the reaction initiation. Interestingly, an investigation of microcrystal flow through capillaries indicated the presence of a depletion zone containing no crystals near the capillary wall, which introduces an intrinsic cutoff of low-speed tails and alleviates flow smearing (Grünbein, 2017 ▸). Alternatively, a flow geometry can be adopted such that the sample fluid is localized near the centreline of the capillary so that all crystals travel at nearly the same flow speed. Both PDMS-based (Trebbin *et al.*, 2014 ▸) and capillary-based (Wang *et al.*, 2014 ▸; Calvey *et al.*, 2016 ▸) mixers have been designed for this purpose. These operate like a ‘jet-in-jet’ system, similar to the double-flow focusing nozzle (Oberthuer *et al.*, 2017 ▸), where the microcrystal suspension is focused by the substrate solution in a first liquid-focusing stage, which initiates the mixing prior to gas focusing and injection into the X-ray interaction region. Depending on the relative position of the liquid- and gas-focusing stages, the flow rate and geometry, a time resolution of a few milliseconds can be achieved (Calvey *et al.*, 2016 ▸). This was exploited to extend the study of ceftriaxone cleavage by β-lactamase to shorter time delays (Olmos *et al.*, 2018 ▸). While the required time for efficient mixing of the substrate into the sample decreases with increasing flow rate of the substrate solution, this also ‘dilutes’ the concentration of crystals in the emitted liquid jet, leading to a decrease in the hit rate and thus increasing the time required to collect a data set (Calvey *et al.*, 2016 ▸).

Despite these first applications of the mixing concept, further experimental characterizations of the various parameters, such as optimal mixing dynamics and diffusion times into crystals, need to be performed.

### 2.2. High-viscosity extrusion (HVE)   

The viscosity of an HVE sample needs to be high enough to allow the extrusion of a slow free-standing sample stream out from the inner sample capillary at low flow rates while gas is flowing in the outer capillary (Fig. 1 ▸ *b*). High pressures are generally required for this, often in excess of those that can be supplied by an HPLC-based liquid-delivery system. A piston-driven sample reservoir must then be installed between the HPLC and the nozzle, with the piston serving both as a pressure amplifier and as the means of separating the sample from the hydraulic fluid (generally water). The high viscosity of an HVE sample precludes GDVN gas focusing, meaning that the extruded free stream is roughly equal in diameter to the inner diameter of the sample capillary, which is typically 50–100 µm. Sample flow rates can be as low as tens of nanolitres per minute; thus, sample efficiency is much higher and sub-milligram sample quantities can be sufficient (Weierstall *et al.*, 2014 ▸; Botha *et al.*, 2015 ▸; Kovácsová *et al.*, 2017 ▸). In HVE operation, the sheath gas flow serves only to direct the sample downstream and to thereby avoid balling up of the sample as it exits the capillary. For HVE injection into a vacuum, the sheath gas also counteracts evaporative cooling of the stream. The initial version of this injector was developed at Arizona State University for SFX of membrane-protein crystals grown in the mesophase lipidic cubic phase (LCP; Weierstall *et al.*, 2014 ▸), and was hence given the appellation ‘LCP injector’. With improvements to the injector by the Heidelberg group, with the introduction of high-viscosity carriers other than LCP and with porting to synchrotron use, the HVE designation became more appropriate (Botha *et al.*, 2015 ▸). A Hamilton syringe-based high-viscosity injector was initially used at SACLA (Sugahara *et al.*, 2015 ▸) without separate pressure amplification.

HVE is also well suited for serial sample delivery at synchrotrons, as the extruded viscous stream is sufficiently slow that the exposed crystal in the stream is essentially still during the relatively long (compared with XFELs) exposure time. Thus, the diffraction patterns are not ‘smeared out’ by crystal movement, as shown recently at the SLS (Botha *et al.*, 2015 ▸; Weinert *et al.*, 2017 ▸), the ESRF (Nogly *et al.*, 2015 ▸) and APS (Martin-Garcia *et al.*, 2017 ▸).

#### 2.2.1. Viscous matrices for HVE   

The suitability of HVE for SFX strongly depends on the viscous matrix in which the macromolecular crystals are immersed. Consequently, a suitable HVE matrix must fulfil a number of requirements. Firstly, it must have the viscosity and flow properties needed to form a stable stream when injected. Secondly, it must be inert to or compatible with the crystals and their mother liquor. Thirdly, it must neither affect the diffraction properties of the crystals nor have a strong X-ray background itself, as the stream is invariably much thicker than the crystals that it carries. Ideally, it should also be compatible with injection into both atmospheric ambient pressure and vacuum (Kovácsová *et al.*, 2017 ▸). To date, several such matrices have been identified and fulfil these requirements to varying extents, usually depending on their chemical nature: whether it is the lipid bilayer forming the LCP or a hydrophilic or hydrophobic system.

LCP is very frequently used for HVE owing to its naturally viscous consistency and the fact that many membrane-protein crystals, such as small and weakly diffracting G-protein-coupled receptor crystals, can be crystallized in it and then directly injected into the intense XFEL beam (Liu *et al.*, 2014 ▸). LCP is a bicontinuous system of lipid bilayers obtained by mixing specific monoacyl glycerols (MAGs; most commonly monoolein, MAG 9.9) with aqueous solutions in specific ratios. For crystallization, the target protein is reconstituted in the lipid bilayers and the addition of precipitants drives crystal formation (Caffrey & Cherezov, 2009 ▸). The crystal-loaded LCP from multiple crystallization setups is then collected, the remaining precipitant is absorbed by adding lipid (Liu *et al.*, 2014 ▸) and/or the viscosity can be adjusted to appropriate injection properties by adding the copolymer Pluronic F-127 (Kovácsová *et al.*, 2017 ▸). In addition, LCP has an acceptable X-ray background and can be used at atmospheric pressure and also in a vacuum. For *in vacuo* use, to prevent evaporative cooling and thereby an unwanted phase transition of the matrix, either the sample is flowed faster (several millimetres per second) or shorter lipids such as MAG 7.9 (Liu *et al.*, 2014 ▸) are added to the sample post-crystallization. LCP can also be used to deliver non-LCP crystallized samples, for example if sample scarcity limits the use of other injection methods. In these cases, crystals can be embedded in LCP post-crystallization (Botha *et al.*, 2015 ▸; Fromme *et al.*, 2015 ▸), although this may not always be possible. Specific chemicals such as MPD or high concentrations of salts and PEGs may not be compatible with the formation of LCP (Cherezov *et al.*, 2001 ▸). While LCP-SFX remains highly successful for *in meso*-grown crystals (Kang *et al.*, 2015 ▸; Zhang *et al.*, 2015 ▸; Zhang, Qiao *et al.*, 2017 ▸; Zhang, Han *et al.*, 2017 ▸), given its delicate and composition-sensitive nature, other viscous matrices for the embedding of non-LCP crystallized samples have also been explored.

The hydrophobic matrices comprise mineral oil-based grease (Sugahara *et al.*, 2015 ▸), Vaseline (Botha *et al.*, 2015 ▸) and nuclear-grade grease (Sugahara *et al.*, 2017 ▸). Hydrophobic matrices are immiscible with aqueous mother liquors and therefore largely circumvent this mother liquor–matrix compatibility issue, making them very versatile in this respect. In particular, mineral oil-based grease has proven to be compatible with numerous protein crystals (Yamashita *et al.*, 2015 ▸; Colletier, Sliwa *et al.*, 2016 ▸; Fukuda *et al.*, 2016 ▸; Edlund *et al.*, 2016 ▸; Suga *et al.*, 2017 ▸). On the other hand, the general drawbacks of these matrices are a relatively high X-ray background, possible dehydration of the sample and sometimes unreliable injection at room temperature (curling and adhesion of the stream to the tip/sides of the nozzle; see Supplementary Fig. S1 in Kovácsová *et al.*, 2017 ▸). Notably, at least for the mineral oil-based grease, these injection difficulties appear not to arise in vacuum.

The undesired high background of hydrophobic matrices drove the search for matrices towards the chemically different class of hydrogels. To date, several of these have been described: agarose (Conrad *et al.*, 2015 ▸), hyaluronic acid (Sugahara *et al.*, 2016 ▸), hydroxyethyl cellulose (Sugahara *et al.*, 2017 ▸), carboxymethyl cellulose (NaCMC; Kovácsová *et al.*, 2017 ▸), Pluronic F-127 (Kovácsová *et al.*, 2017 ▸) and PEG 8 000 000 (Martin-Garcia *et al.*, 2017 ▸). As these are largely composed of water, the background is generally lower than those of hydrophobic matrices. However, their hydrophilic nature and resultant miscibility with mother liquors raises compatibility issues. Consequently, the choice of hydrogel to use for a specific crystalline sample is a matter of testing and optimization. The use of hydrogels in a vacuum is possible with the addition of cryoprotectants such as glycerol (Conrad *et al.*, 2015 ▸). For two hydrogels, NaCMC and Pluronic F-127, the jet speed as a function of flow rate was investigated for various samples (Kovácsová *et al.*, 2017 ▸). The possibility of precisely and reproducibly controlling the jet speed is particularly important for time-resolved experiments (see Section 2.3).

### 2.3. The importance of jet speed for megahertz repetition-rate and time-resolved experiments   

As outlined above, in serial data collection the sample-delivery technique must ensure that pristine sample is present for each X-ray exposure. Moreover, the sample probed by the X-rays must be in the desired state, namely unaltered by the previous pulse and, for time-resolved experiments, also in a specific activated state that is to be probed. In the case of GDVN or HVE injection, the critical parameter to obtain such conditions is the jet speed, which determines the rate at which sample is supplied to the X-ray interaction region.

Interaction of the XFEL pulse with the jet destroys a section of the jet (‘diffraction before destruction’; Chapman *et al.*, 2014 ▸), and in the case of liquid jets even leads to an explosion that opens up a gap within the jet (Stan *et al.*, 2016 ▸). The time needed to replenish this damaged section with pristine material depends on the jet speed and diameter and may limit the maximum repetition rate for XFEL measurements, which would otherwise be determined by the maximal XFEL repetition or detector recording rate. Notably, damage may extend beyond the gap (obvious damage region) owing to shock waves induced by the interaction with the XFEL pulse which propagate upstream, requiring that not only the gap closes in the time between pulses, but also that the shock-wave-affected material has passed beyond the interaction region (Stan *et al.*, 2016 ▸). While this aspect of supplying pristine material has not been an issue at first-generation XFELs running at up to 120 Hz, it becomes a question of the utmost importance at megahertz repetition-rate XFELs, where the jet speed needs to be much higher and extremely carefully controlled to ensure that pristine sample is supplied within time delays as short as 0.22–1 µs between X-ray pulses (Altarelli & Mancuso, 2014 ▸; Schoenlein, 2015 ▸). For these reasons, HVE operating at slow jet speeds cannot take advantage of the megahertz repetition rate. In contrast, GDVN injection can achieve high jet speeds (Grünbein, Shoeman *et al.*, 2018 ▸; Wiedorn, Awel *et al.*, 2018 ▸) and was used for the first experiments exploiting the megahertz repetition rate of hard X-ray pulses (Grünbein, Bielecki *et al.*, 2018 ▸; Wiedorn, Oberthür *et al.*, 2018 ▸). Although no indications of damage were found, the experiments were conducted under ‘mild’ conditions at a low power density owing to a 15 µm X-ray focus. Once the anticipated focus of the SPB/SFX instrument is achieved (1 µm/100 nm; Altarelli, 2011 ▸, 2015 ▸), the question of potential damage needs to be revisited.

Jet speed is also important for optically triggered TR experiments as it can determine the accessible time delays (chemical mixing is covered in Section 2.1.2). After reaction initiation in a given segment of the jet (Fig. 1 ▸ *c*), (i) the very same segment must be in the X-ray interaction region simultaneously with the X-ray pulse and not yet have travelled beyond or not yet have reached it, and moreover (ii) it must have passed beyond the interaction region before the next probe pulse. For ultrafast time delays Δ*t*, the jet is essentially still during Δ*t* and only (ii) needs to be considered. For longer time delays (hundreds of nanoseconds for GDVN, milliseconds for HVE) both points (i) and (ii) matter. Point (ii) sets a minimal jet-speed limit and depends on the optical laser diameter and position. Point (i) sets the maximal and potentially another minimal jet speed and/or the distance between reaction initiation and X-ray interaction. For successful TR measurements, the jet speed must therefore be known and, together with other experimental parameters, adjusted where possible. Generally, the jet speed limits TR measurements in the free jet to a maximal time delay of at most a few microseconds for GDVNs, whereas a few seconds may be achieved with HVE.

#### 2.3.1. Jet-speed measurements   

To measure the jet speed, a feature travelling with the jet at the same speed needs to be tracked over time. For viscous jets, this is relatively easy, since the jet speed is low (of the order of a few millimetres per second) and the displacement of a feature (for example a crystal) can be tracked without difficulties using a camera with a reasonable frame rate and magnification (Kovácsová *et al.*, 2017 ▸). For liquid jets this becomes more problematic owing to the high jet speed (10–100 m s^−1^) and microscopic jet size, which means that tracking a feature over time requires fast time resolution both to prevent blurring and to capture the feature at two or more successive positions. Pulsed (laser) illumination of sufficiently short duration (a few nanoseconds or less) can be used to deliver sharp images of liquid jets (Stan *et al.*, 2016 ▸). To measure the jet velocity of high-speed jets, either a camera of sufficient frame rate (up to one million frames per second) is required in combination with such pulsed illumination or, more conveniently, double exposure of the jet within single frames can be used to extract the jet speed in combination with a (triggerable) camera of arbitrary frame rate (Grünbein, Shoeman *et al.*, 2018 ▸). Similar to exploiting such double-exposure images, the jet speed can be determined from images of the jet hit by multiple consecutive X-ray pulses each producing one gap inside the contiguous segment, so that the distance between gap centres and the known time delay between X-ray pulses can be used to extract the jet speed (Grünbein, Bielecki *et al.*, 2018 ▸; Wiedorn, Awel *et al.*, 2018 ▸).

## 3. Fixed target for serial sample delivery   

Fixed-target techniques are another means of serially delivering fresh sample for each X-ray exposure. Here, the crystals are immobilized on a substrate, which is then scanned through the X-ray beam. An inherent advantage of this approach is that in principle the geometry and crystal distribution can be arranged such that every crystal on the substrate is probed. Each step in a raster scan must clearly move beyond the area affected by previous probe pulses, which is particularly important at an XFEL. Numerous solid-support approaches have been developed over the years and, depending on the design, they mainly vary in (i) the X-ray background, which can be caused by the substrate itself and by excess mother liquor, (ii) the extent to which they support high-throughput data collection in terms of maximal data-collection rate and high hit rate, (iii) whether only specific crystal sizes or shapes can be accommodated, (iv) crystal handling, *i.e.* crystal growth directly on the substrate/off the substrate, preventing crystal dehydration during loading or data collection, and (v) whether they can be used at room or cryogenic temperature.

Goniometer-based approaches using standard loops and micromeshes or specialized high-density sample-mounting grids have successfully been used at XFELs with larger microcrystals (>20 µm) at cryogenic temperature (Cohen *et al.*, 2014 ▸) and also at room temperature (Baxter *et al.*, 2016 ▸). These approaches together with related fixed-target methods are extensively covered by another article in this issue (Martiel *et al.*, 2019 ▸).

To circumvent manual crystal handling, microfluidic chips have been employed for on-chip crystallization either by free-interface diffusion (Perry *et al.*, 2013 ▸) or by using highly controlled water-in-oil emulsions (Heymann *et al.*, 2014 ▸). These were directly used for on-chip X-ray diffraction data collection using synchrotron radiation at room temperature, including serial time-resolved Laue diffraction (Perry *et al.*, 2014 ▸; Pawate *et al.*, 2015 ▸). For XFEL measurements, microcrystals (<15 µm) obtained by off-chip crystallization can be loaded and very efficiently captured in a microfluidic trap array (Lyubimov *et al.*, 2015 ▸). The general drawback of microfluidic chips, however, is the relatively high X-ray background of the polymer (PDMS) and, in some cases, rapid water diffusion into the polymer causing bubble formation and clearing of the traps and channels (Lyubimov *et al.*, 2015 ▸).

Minimizing the X-ray background is critical for collecting data from small and/or weakly diffracting crystals. This can be achieved by depositing crystals into chip-defined windows sealed with a sufficiently thin film to produce little X-ray background (such as polyimide or silicon nitride). These windows can be endowed with modified surface properties (hydrophobicity/hydrophilicity, surface roughness) to promote random crystal orientations within a silicon mesh and thereby provide an efficient sampling of reciprocal space (Zarrine-Afsar *et al.*, 2012 ▸). Similarly, the chips can be manufactured from hydrophilic photoresists to promote the positioning of crystals into windows (Murray *et al.*, 2015 ▸). A different silicon-chip design was used for *in vacuo* experiments, with large windows (200 × 8400 µm) etched into silicon and crystals mixed with Paratone N ‘painted’ onto the remaining 50 nm layer of silicon nitride as a support (Hunter *et al.*, 2014 ▸). Alternatively, crystals can be sandwiched between two thin silicon nitride layers without the need for windows (Coquelle *et al.*, 2015 ▸).

To reduce background scattering even further, silicon chips can incorporate microscopic ‘wells’ (Mueller *et al.*, 2015 ▸; Oghbaey *et al.*, 2016 ▸; Owen *et al.*, 2017 ▸) or micropores (Roedig *et al.*, 2015 ▸, 2016 ▸, 2017 ▸) to trap the crystals as excess mother liquor is removed from the chip surface. In both cases, each chip contains thousands of these features, which are fabricated with diameters of an appropriate size so that the crystals of interest are trapped in the wells/holes as the liquid is blotted/sucked away (Roedig *et al.*, 2015 ▸; Mueller *et al.*, 2015 ▸). Crystal loading is performed in a humidity-controlled environment (Roedig *et al.*, 2015 ▸; Oghbaey *et al.*, 2016 ▸). In the case of the chip with micropores, crystals are randomly positioned on the micropatterned membrane and either flash-cooled for synchrotron data collection (Roedig *et al.*, 2015 ▸) or kept further under a stream of humidified gas to mitigate dehydration during room-temperature data collection at a synchrotron (Roedig *et al.*, 2016 ▸) or an XFEL (Roedig *et al.*, 2017 ▸). In case of the ‘well’ design, the intent is to localize the crystals predominantly in the tapered wells. The loaded chip is sealed with Mylar foil for room-temperature data collection at an XFEL (Mueller *et al.*, 2015 ▸; Oghbaey *et al.*, 2016 ▸) or at a synchrotron (Owen *et al.*, 2017 ▸). Coupled with fast and accurate raster-translation systems (Sherrell *et al.*, 2015 ▸; Roedig *et al.*, 2017 ▸), both chip designs can support high-throughput data collection at first-generation XFELs operating at up to 120 Hz, resulting in several minutes being sufficient for a complete data-set collection and hit rates approaching nearly 100% (Oghbaey *et al.*, 2016 ▸; Roedig *et al.*, 2017 ▸; Owen *et al.*, 2017 ▸). On the other hand, raster-scan chip designs rely on very accurate alignment with the XFEL beam passing exactly through the holes, since hitting the chip material produces undesired silicon diffraction and can also damage the chip beyond reuse. This approach becomes problematic if the experimental conditions require, for example, an X-ray beam size, intensity or photon energy that necessitates holes that are too large to trap the crystals of interest. In this case, one can simply sandwich the crystals between two thin Mylar foils, in a so-called sheath-on-sheath (SOS) sandwich (Doak *et al.*, 2018 ▸). Another advantage of the SOS sandwich is that highly accurate alignment is not needed as there are no features facilitating on-the-fly scanning. The drawback is that since the crystals are randomly distributed and not predominantly located in defined wells the sample efficiency is lower, but it is still of the same order of magnitude as for HVE injection (Doak *et al.*, 2018 ▸).

## 4. Conclusion and outlook   

Despite the increasing variety of sample-delivery techniques, there is no universal technique of choice for serial crystallo­graphic data collection at XFEL or synchrotron sources. Instead, the suitability of a specific technique strongly depends on the investigated system and the experimental aim and conditions. We expect serial sample delivery to remain a rapidly developing field as the number of next-generation synchrotrons and XFELs is increasing, as will the user community.

## Figures and Tables

**Table 1:** A comparison of sample-delivery characteristics using liquid jets, viscous jets and fixed targets Given the rough grouping of the methods and the fact that many of the parameters depend on other experimental settings (such as the crystal shape, mother-liquor composition, crystal symmetry *etc.*, to name a few), this table provides a high-level overview.

Parameters	Liquid jets	Viscous jets	Fixed targets
Sample-translation speed	∼10–100 m s^−1^	Up to several millimetres per second	Defined by motors
Jet diameter	≤5 µm	Defined by the inner diameter of the capillary, ∼50–10 µm	N/A
Flow rate	∼5–50 µl min^−1^	Tens of nanolitres to several microlitres per minute	N/A
X-ray background†	Generally low	Matrix-dependent; generally higher than for liquid jets	Design- and sample thickness-dependent
Suitable crystal size	≤∼10 µm	Smaller than the inner diameter of the capillary	If chip is patterned, feature-dependent; otherwise, any size
Sample consumption	Tens of milligrams or more	Sub-milligram possible‡	Sub-milligram possible‡
Sample efficiency			
At ≤120 Hz repetition rate	Low	High	High
At megahertz repetition rate	High	Unfeasible	High
Beam-time efficiency			
At ≤120 Hz repetition rate	High	High	High
At megahertz repetition rate	High	Repetition-rate decrease necessary	To be seen§
Longest time delay with optical triggering	A few microseconds or less	A few seconds or less	Unrestricted
Suitability for mixing	Yes	No	Yes
Suitability for synchrotron use	Currently not¶	Yes	Yes

† A detailed comparison is difficult as many parameters contribute to the measured background (sample, scattering environment *etc*.) and prevent such a cross-publications comparison. For details, see Sections 2.1, 2.2.1 and 3.

‡ Optically triggered TR experiments require much more sample since not only the X-ray affected section but the entire optically illuminated section of the jet needs to be displaced between the exposures.

§ The challenge will be the acceleration and deceleration times of linear scanning. This may be eliminated with radial scanning.

¶ May be feasible at diffraction-limited storage rings. The exposure must be short enough to capture the fast-moving crystal as essentially ‘still’ and must contain a sufficient number of photons for high-resolution diffraction.
